# The albatross plot: A novel graphical tool for presenting results of diversely reported studies in a systematic review

**DOI:** 10.1002/jrsm.1239

**Published:** 2017-04-28

**Authors:** Sean Harrison, Hayley E. Jones, Richard M. Martin, Sarah J. Lewis, Julian P.T. Higgins

**Affiliations:** ^1^ School of Social and Community Medicine University of Bristol Bristol UK; ^2^ MRC Integrative Epidemiology Unit (IEU) at the University of Bristol Bristol UK

**Keywords:** evidence synthesis, graphical tool, methodology, systematic review

## Abstract

Meta‐analyses combine the results of multiple studies of a common question. Approaches based on effect size estimates from each study are generally regarded as the most informative. However, these methods can only be used if comparable effect sizes can be computed from each study, and this may not be the case due to variation in how the studies were done or limitations in how their results were reported. Other methods, such as vote counting, are then used to summarize the results of these studies, but most of these methods are limited in that they do not provide any indication of the magnitude of effect.

We propose a novel plot, the albatross plot, which requires only a 1‐sided *P* value and a total sample size from each study (or equivalently a 2‐sided *P* value, direction of effect and total sample size). The plot allows an approximate examination of underlying effect sizes and the potential to identify sources of heterogeneity across studies. This is achieved by drawing contours showing the range of effect sizes that might lead to each *P* value for given sample sizes, under simple study designs. We provide examples of albatross plots using data from previous meta‐analyses, allowing for comparison of results, and an example from when a meta‐analysis was not possible.

## 1 INTRODUCTION

Meta‐analyses combine the results of multiple studies of a common research question. They typically focus on estimation of an underlying (average) effect size across studies, and often illustrate the individual study results and the pooled result using a forest plot. Meta‐analyses of this type are, however, not always possible. This is especially the case when data are collected, analysed, or reported in different ways in different studies. Sometimes statistical analysis results are presented in ways that do not facilitate estimation of a comparable effect size for every study. One possibility in this situation is to perform a narrative synthesis of the findings across studies. This can be cumbersome to digest and there is a risk that conscious or unconscious bias may affect the way in which the results are presented.

Where statistical test results are available from each study, vote counting might be used, in which the numbers of studies reporting a positive, negative or null association using a predefined *P* value threshold are counted. Harvest plots have been proposed as an extension of vote counting, providing a graphical tool for displaying the results from each study.^1^ In a harvest plot, each study is represented by a bar whose height and appearance convey information related to confidence in the result (eg, study design), and the bars are grouped by whether the study found a positive, negative, or null association. The practice of distinguishing between “significant” and “nonsignificant” findings has lost favour in recent years.^2^ Some specific limitations of drawing this distinction using vote counting approaches to meta‐analysis are that they do not account for differences in the relative sizes of the studies and do not provide measures of the magnitude of any effects. They are also problematic when studies are small, since statistically significant results can be difficult to obtain even when effect sizes are reasonably large. The vote counting approach has been widely criticized for these reasons.^3^, ^4^

More attractive alternatives to vote counting are available. One possibility is a sign test, in which directions of effect are counted rather than conclusions around statistical significance. A second possibility is the statistical combination of exact precise *P* values across studies, for example, using methods of Fisher^5^ or Stouffer.^6^ These approaches both produce an overall *P* value for testing the null hypothesis of no effect in every study. However, again, they take no account of the relative sizes of the studies and do not provide an estimate of effect magnitude.

In this paper, we propose a novel plot to illustrate findings from quantitative studies when insufficient information is available to present results in a forest plot. Our albatross plot is based on minimal statistical information that is usually available from each study, namely, a precise *P* value and a total sample size. However, unlike the simple methods described above, albatross plots allow an approximate examination of underlying effect sizes and the potential to identify sources of heterogeneity across studies.

We introduce the albatross plot in Section 2, and explain how we illustrate approximate effect sizes using superimposed contours in Section 3. In Section 4, we illustrate the albatross plot using four example data sets: the first is a meta‐analysis of randomized trials of exercise training after acute myocardial infarction (MI), which had originally been analysed using effect sizes; the second is a meta‐analysis of correlations between student ratings of college professors and their achievement levels, which has been used to illustrate meta‐analysis of *P* values; the third is from a systematic review of the association between milk intake and insulin‐like growth factor‐I (IGF‐I), where a meta‐analysis was not possible because of the diverse reporting of studies; and the fourth is from a review of the association between body mass index (BMI) and prostate specific antigen (PSA), where a meta‐analysis was possible but not for all studies.

## 2 THE ALBATROSS PLOT

Appropriate interpretation of *P* values requires information about the sizes of the study from which they come. For example, a *P* value of 0.2 may arise from a large study with a small underlying effect or from a small study with a large underlying effect. Our basic albatross plot is a scatter plot of study sample sizes against 1‐sided *P* values. Equivalently, this is a scatter plot of study sample sizes against 2‐sided *P* values, with results separated according to the observed direction of effect. The albatross plot allows the *P* values to be interpreted in the context of the study sample size. Small studies appear towards the bottom of the plot and large studies towards the top. Throughout this paper, we plot 2‐sided versions of the *P* values, separated by direction of effect, because these are much more commonly reported. One‐sided *P* values can readily be transformed to 2‐sided *P* values.

Small 2‐sided *P* values from strong negative results (eg, corresponding to 1‐sided *P* values near 0) appear at the left of the plot and small 2‐sided *P* values from strong positive results (corresponding to 1‐sided *P* values near 1) appear at the right of the plot, with studies with null results towards the middle. We plot both the sample size axis and the *P* value axis on the log scale for improved visual interpretation.

Two types of enhancement to the basic albatross allow approximate examination of effect sizes and their heterogeneity. First, we superimpose contours on the plot to reflect different hypothetical effect sizes that would have given rise to particular *P* values. These contours will be specific to the type of data (and statistical methods) used to calculate the *P* values and are to be interpreted very approximately. The contours typically resemble large flying birds, giving rise to our proposed name of an albatross plot. We describe some simple derivations for contours in the following section and derive a variety of other possibilities in an online supplement. Second, different subgroups of studies can be drawn using different colours or symbols to facilitate identification of subgroup effects.

**Figure 1:**
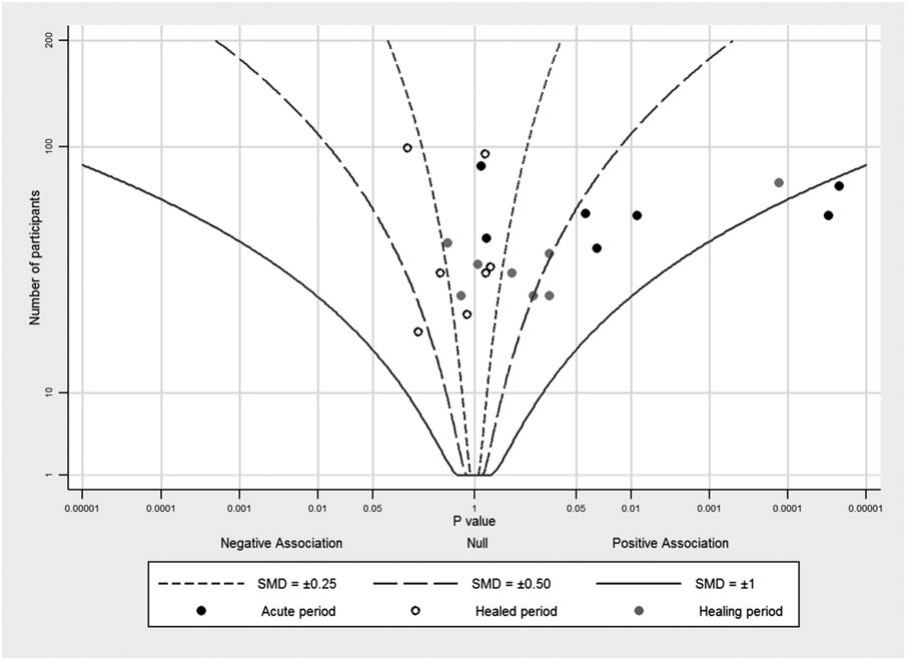
Albatross plot for studies of the effect of exercise training on left ventricular fraction after acute myocardial infarction, with contours for standardized mean differences (SMDs), using data from Zhang et al^11^

**Figure 2:**
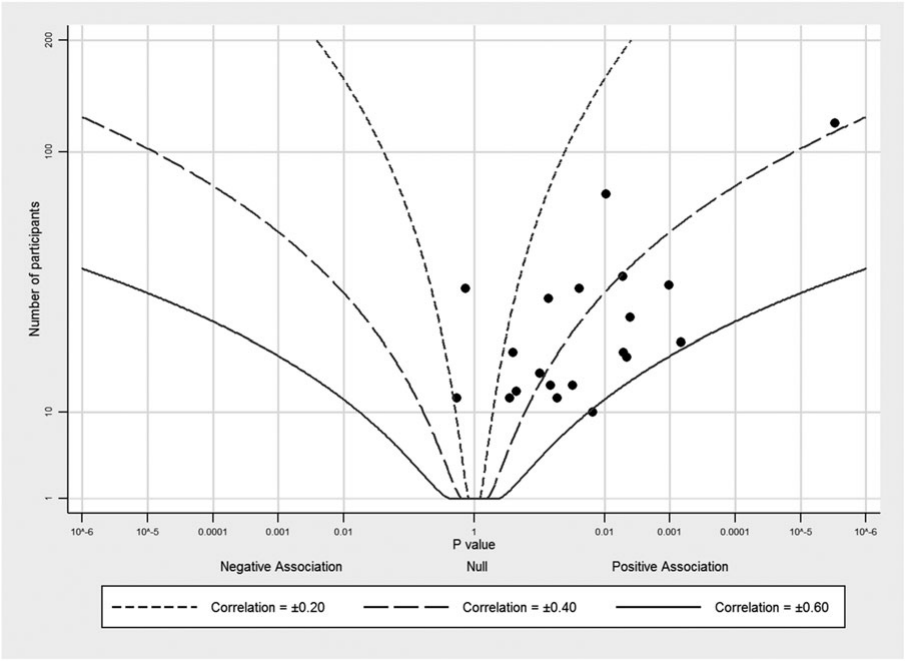
Albatross plot for studies of student ratings of their college instructors and student achievement levels with contours for correlation coefficients, using data from Becker^14^

**Figure 3:**
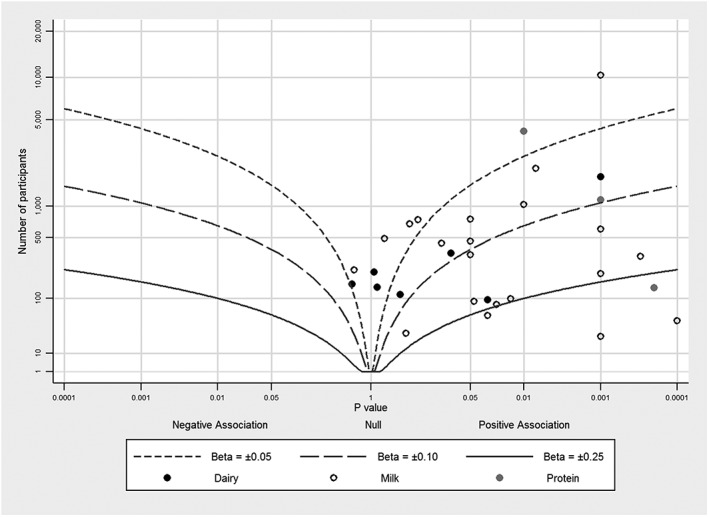
Albatross plot for studies of the association between milk intake an insulin‐like growth factor‐I, using data from Harrison et al (Harrison et al, In press meta‐analysis, 2016)^15^

**Figure 4:**
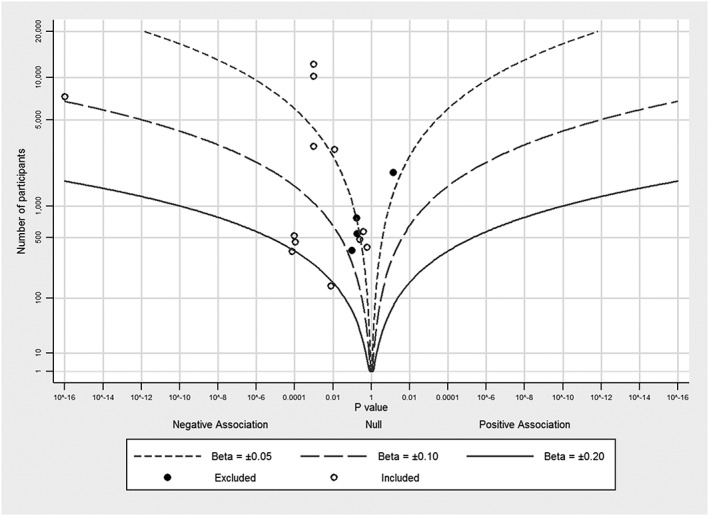
Albatross plot for the association between body mass index and prostate specific antigen, using data from Harrison et al (Harrison et al, unpublished meta‐analysis, 2016)^16^

Figures 1, 2, 3, 4 provide examples of albatross plots, and we discuss these in more detail in Section 4. It is visually clear if the studies generally convey a positive or a negative effect size, since the studies will cluster on one side of the plot. If the studies have generally a similar effect size, the points will fall around an effect size contour; if there is heterogeneity of effect size, then the points will be scattered across contours reflecting heterogeneous effect sizes. If there is no association, points will fall evenly around the null in the centre of the plot. Smaller studies will have larger variation around the true value and will have points that are more clustered around the null than larger studies. However, many small studies can still point towards a single effect size contour if the underlying effects are homogeneous. Furthermore, outlying studies can be identified with ease, and a brief narrative synthesis conducted alongside the albatross plot might propose possible explanations for the findings in these studies.

## 3 EFFECT SIZE CONTOURS

Our approximate effect size contour lines are based on the general assumption that the *P* values were derived from Wald tests. A Wald test involves division of the effect size estimate (*b*) by its standard error (SE) to calculate a *Z*‐statistic

(1) $$Z_{P}=\frac{b}{\mathrm{SE}}.$$

This statistic is compared to a standard normal distribution to obtain the *P* value. Conversely, a *Z*‐statistic can be obtained from a (reported) *P* value using the same distribution, so we write *Z*_*p*_ ≡ Φ^−1^(*P*), where Φ^−1^ denotes the inverse of the standard normal distribution function.

In general, the SE is proportional to the inverse of the square root of the total number of participants (N) in the study, so that we can write

(2) $$\mathrm{SE}=\frac{\phi }{\sqrt{N}}.$$

The quantity *ϕ* may be a fixed number, or it may involve the effect size itself, and it may additionally involve other quantities that need to be specified to define an effect size contour uniquely.

Rearranging Equations (1) and (2), we can express the sample size in the form

(3) $$N=\frac{\phi^{2}}{b^{2}}Z_{P}^{2}.$$

Since *Z*_*p*_ has a one‐to‐one correspondence with the horizontal axis in the albatross plot, to obtain a contour corresponding to a hypothetical effect size *b*, we need only determine the quantity *ϕ* appropriate for the choice of effect size, and plot *N* as a function of *ϕ*, *b*, and *P*.

In Section 3.1, we illustrate the derivation of contours for standardized mean differences (SMDs). We then use simple relationships between SMDs, odds ratios (ORs), and correlation coefficients to produce crude contours for these other two measures. In Data S1, we show how effect size contour lines can be derived more exactly for ORs and correlation coefficients, as well as for a variety of other effect measures that are commonly encountered in meta‐analysis, including mean differences, risk ratios, and regression coefficients. We provide the formulae that define contours for these effect measures in Table 1. Stata code to generate an albatross plot, with a help file and examples, is available to download from the SSC using the Stata package name albatross (type “ssc install albatross” in Stata to download).

**Table 1:** Formulae for calculating effect size contours for different effect measures

Effect measure	Equation	Additional variables requiring values
Mean difference (MD) equal sized groups	$N=\frac{4{\mathrm{SD}}^{2}}{{\mathrm{MD}}^{2}}Z_{p}^{2}$	Standard deviation (SD)
Mean difference (MD) unequal sized groups	$N=\frac{{\mathrm{SD}}^{2}{\left(r+1\right)}^{2}}{{r\times \mathrm{MD}}^{2}}Z_{p}^{2}$	Standard deviation (SD) Ratio of group sizes (*r* = *n*_1_/*n*_2_)
Standardized mean difference (SMD) equal sized groups	$N=\frac{8+{\mathrm{SMD}}^{2}}{{2\mathrm{SMD}}^{2}}Z_{P}^{2}$	(none)
Standardized mean difference (SMD) unequal sized groups	$N=\frac{{2\left(r+1\right)}^{2}+r\times {\mathrm{SMD}}^{2}}{{2r\times \mathrm{SMD}}^{2}}Z_{p}^{2}$	Ratio of group sizes (*r* = *n*_1_/*n*_2_ )
Correlation coefficient (*ρ*)	$N=\frac{1-\rho^{2}}{\rho^{2}}Z_{p}^{2}$	(none)
Standardized beta coefficient (*β*_*s*_) from univariable linear regression	$N=\frac{1-\beta_{s}^{2}}{\beta_{s}^{2}}Z_{p}^{2}$	(none)
Odds ratio (OR) equal sized groups	$N=\frac{2\left[{\left(1-\pi_{2}+\pi_{2}\text{OR}\right)}^{2}+\text{OR}\right]}{\pi_{2}\left(1-\pi_{2}\right)\times \text{OR}\times {\left(\ln\text{OR}\right)}^{2}}Z_{p}^{2}$	Control group risk (*π*_2_)
Odds ratio (OR) unequal sized groups	$N=\frac{\left(r+1\right)\left[{\left(1-\pi_{2}+\pi_{2}\text{OR}\right)}^{2}+r\text{OR}\right]}{{\mathrm{r\pi}}_{2}\left(1-\pi_{2}\right)\times \text{OR}\times {\left(\ln\text{OR}\right)}^{2}}Z_{p}^{2}$	Control group risk (*π*_2_) Ratio of group sizes (*r* = *n*_1_/*n*_2_)
Risk ratio (RR) equal sized groups	$N=\frac{2\left(1+\mathrm{RR}-2\mathrm{RR}\times \pi_{2}\right)}{\pi_{2}\times \mathrm{RR}\times {\left(\ln\mathrm{RR}\right)}^{2}}Z_{p}^{2}$	Control group risk (*π*_2_)
Risk ratio (RR) unequal sized groups	$N=\frac{\left(r+1\right)(r+\mathrm{RR}\left(r-\pi_{2}-r\pi_{2}\right)}{r\pi_{2}\times \mathrm{RR}\times {\left(\ln\mathrm{RR}\right)}^{2}})Z_{p}^{2}$	Control group risk (*π*_2_) Ratio of group sizes (*r* = *n*_1_/*n*_2_)

N = total number of participants (*N* = *n*_1_ + *n*_2_) in two‐group studies.

***Z***_***P***_ = *Z* value for the associated 2‐sided *P* value; **Φ**^−1^(***P***) ≡ ***Z***_***p***_.

Contours for all effect measures require specification, by the user, of the effect size to which the contour relates. For some effect sizes, including the SMD for a study with equal group sizes, this effect size is all that needs to be specified to determine *ϕ*. In other cases, further variables must be specified, such as the ratio of group sizes or the baseline risk. Values for the additional variables might be chosen using the most common values in the included studies; for instance, for trials the ratio of group sizes is often 1, so there are equal number of participants in the intervention and control arms of the trial.

Some studies do not report explicit *P* values but state that *P* is more than or less than a threshold value, for example, *P* < 0.05. Where possible, a precise *P* value should be calculated from data. If this is not possible, for *P* < threshold, we suggest either assuming *P* = threshold (eg, *P* = 0.05) or drawing a line instead of a point on the albatross plot to show the range of *P* values compatible with the reported finding (eg, a line between *P* = 0.05 and *P* = 0.01). For *P* > threshold, we suggest that either omitting the study or again using a line to show the range of *P* values compatible with the reported finding (eg, a line between 1 and 0.05).

### 3.1 Effect size contours for SMDs

A simple and commonly used effect size is the SMDs, which compares mean responses between two groups, such as an intervention group and a control group in a randomized trial. We will use this effect measure as an example of how we create effect contours for the albatross plot.

The SMD is defined as

(4) $$\mathrm{SMD}=\frac{\mu_{1}-\mu_{2}}{\mathrm{SD}},$$

where *μ*_1_ and *μ*_2_ are the mean responses in the two groups of the study and SD is the standard deviation of responses. A simple estimate (Cohen's *d*) of the SMD is obtained by substituting estimates of the means and the pooled SD into Equation (4). An approximate standard error for this estimated SMD is

$$\mathrm{SE}=\sqrt{\frac{1}{n_{1}}+\frac{1}{n_{2}}},$$

where *n*_1_ and *n*_2_ are the sample sizes in the two groups, such that *N* = *n*_1_ + *n*_2_. If the two groups are the same size, then $\mathrm{SE}=\frac{2}{\sqrt{N}}$ . Hence *ϕ* is simply equal to 2, and the effect size contour for each specific value of SMD is obtained as

$$N=\frac{4}{{\mathrm{SMD}}^{2}}Z_{P}^{2}.$$

A better approximation^3^ to the SE of the SMD is

(5) $$\mathrm{SE}=\sqrt{\frac{1}{n_{1}}+\frac{1}{n_{2}}+\frac{{\mathrm{SMD}}^{2}}{2\left(n_{1}+n_{2}\right)}}.$$

Again assuming equally sized groups, we obtain

$$\phi =\sqrt{\frac{{8+\mathrm{SMD}}^{2}}{2}},$$

such that the contours are defined by

(6) $$N=\frac{8+{\mathrm{SMD}}^{2}}{{2\mathrm{SMD}}^{2}}Z_{P}^{2}.$$

Thus, the effect contour for a given SMD, which shows the number of participants required for a particular *P* value, does not require any additional information to create. For example, for an SMD of 0.1, a *P* value of 0.05 (*Z* = 1.96) would arise from a study of 1539 participants.

The assumption of equal sample size in both groups may be a reasonable approximation for experimental studies such as randomized trials but may not be appropriate for observational studies. To account for unequal group sizes when the ratio varies across studies, it is possible to adjust the plotting position for individual points to reflect an effective sample size rather than the observed sample size. Such adjustments to the sample size may require assumptions to be made about the magnitude of the effect size. In our experience, the effect of this adjustment is typically minimal.

While it is possible to use the contour generation equations to estimate the effect size and SE of each study, this is not the purpose of the equations, nor is it advisable for all studies. Rather, the equations define hypothetical effect contours that can aid in interpretation of the magnitude of effect across many studies and are useful for when there is insufficient information to calculate effect sizes and SEs for all included studies.

### 3.2 Crude effect size contours for ORs and correlation coefficients

Under particular assumptions, ORs and correlation coefficients can be transformed to and from an SMD.^3^, ^7^, ^8^, ^9^ Thus, the contours described above for SMDs can be used to provide contours for these measures. To obtain crude contours for the OR, we can substitute the approximation

$$\mathrm{SMD}=\frac{\sqrt{3}\times \ln\text{OR}}{\pi }$$

into Equation (6) to obtain

$$N=\frac{8\pi^{2}+3\;{\ln\text{OR}}^{2}}{6{\ln\text{OR}}^{2}}Z_{P}^{2},$$

where lnOR is the (natural) logarithm of OR.

Similarly, using the approximation for correlation coefficients (*ρ*),

$$\mathrm{SMD}=\frac{2\rho }{\sqrt{1-\rho^{2}}},$$

we obtain

(7) $$N=\frac{2-\rho^{2}}{2\rho^{2}}Z_{P}^{2}.$$

Correlation coefficients are equivalent to standardized regression coefficients (sometimes referred to as betas) from univariable linear regression,^10^ so Equation (7) can be used for standardized regression coefficients, with *ρ* substituted by beta.

## 4 APPLICATIONS

### 4.1 Example 1: randomized trials of exercise training

**Figure 5:**
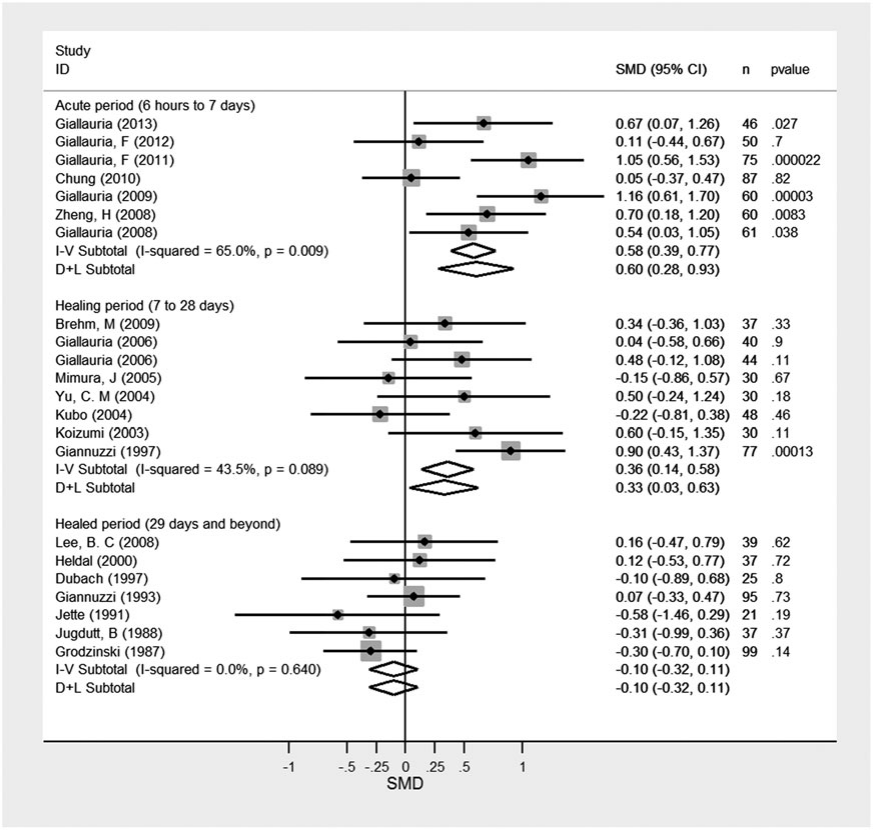
Forest plot of studies of the effect of exercise training on left ventricular fraction after acute myocardial infarction, data from Zhang et al.^11^ I‐V subtotals represent fixed effect meta‐analyses; D+L subtotals represent random effect meta‐analyses. I‐squared is a relative measure of heterogeneity in relation to total variability within each subgroup. SMD, standardized mean difference

To demonstrate the connections between an albatross plot and established techniques, we first illustrate the albatross plot using a data set that was originally analysed using effect sizes and which could, therefore, be illustrated in a forest plot. Zhang et al performed a meta‐analysis of randomized trials evaluating the effect of exercise training after an acute MI.^11^ They examined the endpoint of left ventricular function (LVEF), subgrouping the trials by the period after the MI during which exercise training was initiated. We reproduce the results in a forest plot in Figure 5. In standard random‐effects meta‐analyses, 7 studies initiating training in the “acute” period (6 hours‐7 days) gave mean SMD = 0.60 (95% confidence interval 0.28 to 0.93), 8 studies initiating during the “healing” period (7‐28 days) gave mean SMD = 0.33 (0.03 to 0.63), and 7 studies initiating during the “healed” period (29 days and beyond) gave mean SMD = −0.10 (−0.32 to 0.10). There is evidence of heterogeneity across studies for the first subgroup and to a lesser extent also in the second subgroup but not in the third subgroup. It is, however, evident from these results that exercise training has greater benefit, on average, the earlier it is started.

The albatross plot for these studies is presented in Figure 1, based on *P* values and total sample sizes from the contributing studies. The SMD effect size contours are generated using Equation (6), assuming equal numbers of participants in the exercise training and control group. Different plotting symbols correspond to the three subgroups. For studies in which exercise training started in the acute period, results are clustered to the right side of the plot, showing an improvement in LVEF. The points are centred to the right of the effect size contour with magnitude 0.50, close to the value of 0.60 obtained in the random‐effects meta‐analysis of SMDs. However, heterogeneity among these studies is clear: although each study has a similar sample size, the *P* values are spread horizontally along the graph.

For studies in which exercise training started during the healing period, the points are mostly clustered around an SMD of 0.25. The exception^12^ is separated from the other studies, corresponding to the large SMD of 0.9 observed in this study. For the third subgroup of studies in the healed period group, points are clustered around the null or a little to the left side of the graph, showing no improvement or a little detriment in LVEF, and reflecting the meta‐analysis summary SMD of −0.11.

### 4.2 Example 2: correlation between student ratings of their college instructors and student achievement levels

Our second example uses data from Cohen,^13^ as presented and discussed by Becker in her text about methods for combining *P* values.^14^ Cohen examined the correlation between student ratings of their college instructors and student achievement levels. Becker implemented different methods for combining *P* values, producing combined *P* values between 1.99 × 10^−4^ (using a “minimum *P* value” approach) and 1.25 × 10^−16^ (using Stouffer's *Z*). These tests all demonstrated that student ratings of instructors and achievement levels were correlated. Indeed, a meta‐analysis of the correlation coefficients gave an average correlation of 0.36 (obtained via the weighted average of Fisher *Z*‐transformed correlation coefficients), with no evidence of heterogeneity.

We provide the albatross plot for these *P* values and corresponding study sample sizes in Figure 2. Effect size contours are based on Equation (7). The majority of studies fall between the 0.2 and 0.6 correlation contours, consistent with the observed average of 0.36. Most studies have similar sample sizes, with between 10 and 100 participants. The points, however, have some noticeable scatter across different contour lines. For large sample sizes, this would reflect heterogeneity, whereas for these relatively small sample sizes, it likely reflects mainly sampling error.

### 4.3 Example 3: association between milk intake and IGF‐I

Our third example uses data from a review of studies investigating associations between intake of milk products and IGF‐I, a protein found in blood (S. Harrison et al., In press meta‐analysis, 2016)^15^. In total, 28 studies (with 31 data points) examined this association, but meta‐analysis was not possible because highly diverse reporting, which often lacked sufficient information for calculation of comparable effect sizes and standard errors. The exposure groups have been subdivided into milk, dairy, and dairy protein.

The albatross plot for these studies is presented in Figure 3. Effect size contours are drawn corresponding to several hypothetical standardized regression coefficients using Equation (7), where standardised beta = 1 would be a 1 SD increase in outcome for a 1 SD increase in exposure. The majority of studies show a positive effect size; most studies fall between the standardised beta coefficient contours of 0.05 and 0.25, with the average magnitude of association likely around standardised beta = 0.1. We interpreted this as a small positive association between milk and IGF‐I. The dairy subgroup showed a slightly smaller magnitude of association (around 0.05 SD) than milk; the protein subgroup showed a similar association as milk, but there were only three studies in this group.

As a meta‐analysis was not possible for this review, the albatross plot was useful in determining the likely magnitude of association between milk intake and IGF‐I, as well as in graphically representing all data.

### 4.4 Example 4: association between BMI and PSA

Our fourth example uses data from a review of studies investigating the association between BMI and PSA, a protein found in blood often used as a screening test for prostate cancer (S. Harrison et al., unpublished meta‐analysis, 2016)^16^. In total, 10 studies were included in the meta‐analysis, and 4 additional studies did not have sufficient data for inclusion. An albatross plot was created as a sensitivity analysis to determine if the excluded studies were consistent with the included studies: if the excluded studies fall around or near the included studies, then there is no reason to suspect the inclusion of these studies would materially change the outcome of the meta‐analysis.

The albatross plot for these studies is presented in Figure 4, showing which studies were included in the meta‐analysis and which were not. Effect size contours are drawn corresponding to several hypothetical standardized regression coefficients using Equation (7). All included studies showed a negative effect, with an average magnitude of association likely around standardised beta = −0.05, representing a small negative association between BMI and PSA. Three of the four excluded studies are consistent with the included studies; one excluded study is not as it shows a small positive association. One explanation for this may be that while this study's population was predominantly African men, all other studies had Caucasian and Asian populations.

In this example, the albatross plot was useful in determining that the excluded studies were broadly consistent with the included studies so that, had they been included in the meta‐analysis, the effect estimate would have been unlikely to change. However, the plot also identified one inconsistent study. This identification allowed us to consider possible explanations of the difference and to qualify the results, which may not be generalizable to all populations, appropriately.

## 5 DISCUSSION

Albatross plots provide a versatile and simple way of graphically displaying data from multiple studies when meta‐analysis is not feasible. The basic requirements of the plot are minimal, since it needs only a 1‐sided *P* value and a sample size from each study (or equivalently, a 2‐sided *P* value, a direction of effect, and a sample size). A single plot can show a large amount of information, including multiple results per study. Since most studies will report the number of participants and a *P* value, albatross plots can be more inclusive than a meta‐analysis. Should some studies be excluded from a meta‐analysis due to insufficient information, an albatross plot can be used as a sensitivity analysis to determine whether the excluded studies were consistent with the studies with sufficient information.

Heterogeneity can be seen in the spread of points across the plot, and outliers easily identified. Our proposed effect size contours give a broad indication of the magnitude of an association, while different plotting colours and symbols allow for subgroups to be compared informally. As albatross plots are not designed to estimate the magnitude of an association precisely, the effect size contours and any association seen must be interpreted as approximate; precise combined effect estimates can only be obtained through meta‐analysis. A narrative synthesis may be needed to supplement the plot to fully explore the data, especially for outlying studies.

## CONFLICT OF INTEREST

The authors declare no conflict of interest.
